# Editorial: Bridging science and policy for animal health surveillance: ICAHS4 2022

**DOI:** 10.3389/fvets.2023.1285992

**Published:** 2023-09-26

**Authors:** Lis Alban, Victoria J. Brookes, Fernanda Dórea, Carola Sauter-Louis, Salome Dürr

**Affiliations:** ^1^Department for Food Safety, Veterinary Issues and Risk Analysis, Danish Agriculture & Food Council, Aarhus, Denmark; ^2^Department of Veterinary and Animal Sciences, University of Copenhagen, Frederiksberg, Denmark; ^3^Sydney School of Veterinary Science, University of Sydney, Camperdown, NSW, Australia; ^4^Department of Disease Control and Epidemiology, National Veterinary Institute, Uppsala, Sweden; ^5^Institute of Epidemiology, Friedrich-Loeffler-Institut, Greifswald, Germany; ^6^Veterinary Public Health Institute, Vetsuisse Faculty, University of Bern, Bern, Switzerland

**Keywords:** surveillance, methodology, animal health, food safety, decision-making, policy

The ICAHS4 conference took place in Copenhagen, Denmark, in May 2022, 2 years later than planned due to the COVID-19 pandemic. The conference provided an opportunity for meetings, learning and sharing between all stakeholders involved in surveillance and control of animal health and food safety issues, across sectors such as government, academia and livestock industries.

The COVID-19 pandemic has taught the society the importance of surveillance and early detection of infectious diseases. We have learnt that it is insufficient simply to act on disease emergence and spread. Instead, focus is needed on prevention, surveillance, and early detection of the precursors of emerging infectious disease. Since budgets are limited and the challenges plenty, it is a necessity to collaborate across sectors—academia, governments, industry and the public—in a transdisciplinary way.

Globalization has created a situation where animals are transported across long distances to ensure economic productivity, and foods are traded internationally to keep prices low for consumers. The downside is that with movements of people and goods, hazards may also travel unnoticed, leading to unwanted events. The ongoing spread of African swine fever (ASF) shows the challenges of risk mitigation not only in domestic animals, but also in wildlife. The culling of all mink in Denmark in November 2020 due to fear of spreading of COVID-19 virus resulted in thousands of livestock producers suddenly faced with their life's work disappearing. In addition, the development of antimicrobial resistance (AMR) and the spread of zoonotic pathogens from one part of the world to another demonstrate that the challenges in veterinary public health are global.

To combat these threats, veterinary authorities are under increasing pressure to effectively allocate resources for animal health surveillance and associated risk mitigation; therefore, it is critical to understand why, where and which actions are needed to prevent new threats to animal and public health. Additionally, socio-economic factors influence how actions taken by authorities or livestock industries are perceived by the public. Lack of public involvement may lead to poor understanding and lack of support; for example, vandalism of fences erected in forests to stop ASF from spreading in wild boar. The way forward demands dynamic solutions. Prioritization and feasibility will differ between countries, dependent on local context as well as economic and social values. Therefore, we require global, transdisciplinary collaboration to mitigate global threats, and it is critical to learn from each other to achieve successful prevention, control and mitigation.

This Research Topic contains a selection of the work presented at the ICAHS4 conference, covering the latest experiences in novel research within surveillance for animal health and food safety and security. The intention was to inspire the development of new ways of collaboration; for example, through Public-Private-Partnerships, and interdisciplinary or transdisciplinary approaches. Many novel collaboration models were demonstrated at the conference which allowed participants to learn from each other regarding implementation in practice. Such alternative governance models may lead to cost-effective and successful collaborations.

The areas covered include:

- Surveillance for epidemics and emerging diseases.
- Cross-sector and One Health surveillance.
- Translating surveillance outcomes into policy, decisions and actions.
- Surveillance data.
- Integrating novel methods in surveillance.

The Research Topic consists of 16 original contributions: nine original research articles (including one methods article), four brief research reports, two perspective contributions and one mini-review. The contributions report work undertaken in Denmark, Italy, Lithuania, the Netherlands, Scotland, Spain, Sweden, Thailand, The United States of America, or by international institutions like FAO and WOAH as well as international networks.

Four papers investigate surveillance for epidemics and emerging diseases. Gao et al. focused on the role of empty livestock vehicles returning to Denmark after exports of pigs for the introduction of ASF. Analyses of strengths, weaknesses, opportunities, and threats (SWOT) were conducted related to export of livestock and in particular, of pigs. It was concluded that washing and disinfection, as required and undertaken at the designated stations, are the most important among all risk-reducing measures identified. Hinjoy et al. studied risk perceptions regarding avian influenza among poultry farmers and traders in three border provinces of Thailand adjacent to Laos. According to the 346 respondents' answers, experience in poultry farming was associated with greater risk perception. Regular training could be a way to improve risk perception, and experienced poultry farmers and traders could be part of a community mentorship program to share their experiences and knowledge on avian influenza. Žigaitė et al. evaluated the passive surveillance of SARS-CoV-2 in mink farms in Lithuania. The results showed a prevalence of 23% viral RNA-positive mink farms, and that 84% of the mink farms had been exposed to the virus. The widespread exposure of mink farms to SARS-CoV-2 suggests that passive surveillance is ineffective for early detection of SARS-CoV-2 in mink. Arede et al. described surveillance activities for anthrax, brucellosis, Crimean Congo hemorrhagic fever, foot-and-mouth disease, lumpy skin disease, and peste des petits ruminants that are present or threaten to emerge in the region Black Sea Basin, which consists of Armenia, Azerbaijan, Belarus, Bulgaria, Georgia, Moldova, Romania, Türkiye, and Ukraine. It was concluded that there is a need for stronger international partnerships and resources to strengthen veterinary health capacity, protect animal health and improve ruminant production.

Two papers research cross-sector and One Health surveillance. Moya et al. explored government veterinarians' perception of routine biosecurity in livestock production systems in Spain. The respondents stressed the limited availability of staff and time. The veterinarians interviewed considered that farmers only implement biosecurity measures to avoid being sanctioned, and not because they are aware of the importance of biosecurity. Alban et al. reported from an international network called CoEvalAMR, which is developing guidelines for selection of tools for evaluation of integrated AMU and AMR. Moreover, evaluation tools are systematically assessed using a methodology with a focus on user's experience. Hereby, tool users can share their experience, assisting other users in identifying the most suited tool for their evaluation purpose.

Two papers explore ways of translating surveillance outcomes into policy, decisions, and actions. de Vos et al. described a rapid incursion risk assessment tool for multiple livestock diseases, including the main sources for incursion, and the changes in risk over time. The tool calculates a semi-quantitative risk score for the incursion risk of each disease, and the results enable prioritization. Scollo et al. reported a semi-quantitative risk assessment methodology, developed to classify Italian pig farms in terms of the probability of introduction of ASF, based on farm data collection. The estimation of frequency and levels of non-compliance with biosecurity measures was used to identify weak points in risk prevention at farm level.

Four papers investigate analysis of surveillance data. Schrag et al. explored a method of benchmarking AMU use in the context of farm-level therapeutic incidence (a proxy for disease incidence), and the outcome of that therapy. Reporting AMU in this format addresses multiple primary questions on recording of disease and AMU, necessary for evaluating on farm antimicrobial stewardship in sufficient details. Keck et al. presented the “Assessment Tool for Laboratories and AMR Surveillance Systems” (FAO-ATLASS), which consists of a surveillance and a laboratory assessment module. FAO-ATLASS allows national authorities to systematically assess their AMR surveillance system in food and agriculture and implement a strategic stepwise approach to improve their systems. Marrana et al. reviewed the Laboratory Twinning Programme created in 2006 by the World Organization for Animal Health (WOAH), to balance the global distribution of veterinary laboratory expertise. The review shows that there has been broad uptake and diversity in the focus of the twinning projects implemented in WOAH Member Countries. The programme would benefit from an evaluation that looks at its outcomes and quantifiable impact in beneficiary countries. Comin et al. raised the question of whether meat inspection data can be used for animal health and welfare surveillance. The results covering Swedish pigs and beef cattle showed that some findings are consistently detected and other less. Moreover, calibration and training activities are necessary to enable correct conclusions and for producers to experience an equivalent likelihood of deduction in payment.

Four papers integrate novel methods in surveillance. Moura et al. described the Vet-AMNet system, which was recently developed to collect and analyze national AMU data in Portuguese dairy farms. Outputs were generated by the Portuguese system using Dutch AMU data. The Vet-AMNet system was validated by comparing these outputs with the Dutch result. Duncan et al. evaluated the functionality of the Scottish Animal Disease Surveillance Center. In this recent evaluation, they developed a new denominator using a combination of agricultural census and movement data, to identify relevant holdings more accurately. This provides information that could help policy makers and surveillance providers make decisions about service provision, as well as evaluate the impact of future changes. Dórea et al. discussed how to design analytical workflows focused on decision support. They conclude that the value of data-driven surveillance depends on a “needs-driven” design approach to data digitalization and information delivery. Finally, Gustafsson et al. described the Swedish National Veterinary Institute's workflows and visualization for epidemiological analysis and dynamic report generation to improve disease surveillance. The workflows are designed to be flexible and adaptable to changing data sources and stakeholder demands, with the goal to create a robust infrastructure for the delivery of actionable epidemiological information.

## Author contributions

LA: Conceptualization, Writing—original draft, Writing—review and editing. VB: Writing—review and editing. FD: Writing—review and editing. CS-L: Writing—review and editing. SD: Writing—review and editing.

